# Successful Extirpation of an Intra-Cranial Tumor

**Published:** 1886-03

**Authors:** F. R. Campbell


### Successful Extirpation of an Intra Cranial Tumor.

Translated from *Bull. della Reale Accad. Med.*, by F. R. Campbell, M. D.

At the November meeting of *La Reale Accademia Medica di Roma*, Durante exhibited a patient from whom he had removed an intra-cranial tumor, this being the second case operated on in the history of surgery, and the first successful case on record.

Chiara Battistelli, aged 35. Patient was of a good constitution and well nourished. The left eye projected from the orbit and was turned outward, but the movements and sight were not impaired. The change in the external appearance of the eye began three months before, but this had been preceded by the following symptoms: Gradual loss of sense of smell, loss of memory, especially memory of names; uncertainty of movements, and a feeling of emptiness in the head. Motion and sensation were unchanged. The character of the patient had undergone a change. Formerly she had been of a lively, happy temperament, but she had become morose, melancholy, and was constantly brooding over her health. Hearing, taste, and the digestive functions were unchanged.

From these symptoms, Durante diagnosed the existence of an intra-cranial tumor pressing upon the olfactory nerves, the anterior lobes of the brain, and penetrating the orbital cavity through its roof. He proposed an operation for the removal of the tumor, and the consent of the patient was obtained.

The operation was performed June 1, 1885. An incision was made, running from internal angle of lids of left eye upward, and toward the left to the hair. The flap of skin being turned down, with hammer and chisel a portion of the frontal bone, about 5 centimeters (2 inches) square, was carefully removed from over the orbit. On removing the internal table, the tumor was exposed and carefully drawn out, when it was found that the pedicle was attached to the dura mater of the left lobe. A prolongation of the tumor had descended into the ethmoid cells. It had also depressed the roof of the orbit without penetrating it, and had compressed the left anterior lobe of the cerebrum. The tumor was a sarcoma about the size of an apple.

Hemostatics were employed, a drainage-tube inserted, and the skin flap replaced. The operation lasted about an hour, under chloroform. There was not much hemorrhage, and the patient awoke after the operation with her speech somewhat impaired. In three days a copious discharge of bloody pus flowed through the drainage-tube. On the fourth day the patient became very weak, felt sleepy, and experienced great confusion of mind. The wound was syringed out with antiseptics, and the patient again improved. On the seventh day the sutures were removed, on the ninth the drainage-tube was withdrawn, and on the fifteenth day the patient went home well; although the sense of smell and memory had not greatly improved. There were no indications of a reproduction of the bone removed, but the eye had resumed its normal appearance.

Durante saw his patient three months afterward. Her mental and moral faculties had been entirely restored, and the sense of smell had gradually returned. The sense of smell was found only on one side, the left olfactory having been destroyed.

The only operation of this sort previously attempted, was made by Amidon, of New York, a few months previous to this; but his patient died of meningitis on the eleventh day.

